# Willingness to utilize cervical cancer screening among Ethiopian women aged 30–65 years

**DOI:** 10.3389/fgwh.2022.939639

**Published:** 2022-08-30

**Authors:** Adugna Alemu Desta, Fikadu Tolesa Alemu, Moges Beya Gudeta, Dejene Edosa Dirirsa, Andualem Gezahegn Kebede

**Affiliations:** Department of Midwifery, College of Medicine and Health Sciences, Salale University, Fitche, Ethiopia

**Keywords:** willingness, cervical cancer screening, Girar Jarso, Ethiopia, North Shoa zone

## Abstract

**Background:**

Cervical cancer screening is a means of detecting cervical cancer early, before it develops, in order to reduce disease mortality and morbidity. When women are screened for cervical cancer between the ages of 30 and 40 years at least one time in their life, their risk of cancer could be decreased by 25–36%. Despite this advantage, cervical cancer screening coverage in Ethiopia is still <2%. As a result, we wanted to see how ready women in the Girar Jarso district, Ethiopia, were to get a cervical cancer test.

**Methodology:**

Community-based cross-sectional study was done using a stratified cluster sampling technique among 855 women aged 30–65 years in the Girar Jarso district, Ethiopia, from 1 June 2021 to 1 September 2021. A pretested and semi-structured interviewer-administered questionnaire was used to collect the data. EpiData management version 4.6 was used to enter data, which was then exported to SPSS version 23 for analysis. Logistic regression analysis was performed, and variables with a *p*-value of <0.05 were taken as statistically significant predictors of the willingness to utilize cervical cancer screening.

**Results:**

Of the 855 women, only 315 (46.7%, CI = 43–50.3) women were willing to be checked for cervical cancer, with 181 (21.2%) women having been screened at least one time in their life. Age of 30–39 years [AOR = 2.80 (95% CI: 1.05, 7.48)], urban resident [AOR = 2.12 (95% CI: 1.06, 4.48)], positive attitude [AOR = 1.68 (95% CI: 1.11, 2.53)], wealth status, awareness of cervical cancer, and low perceived barriers were independent predictors of the willingness to utilize cervical cancer screening.

**Conclusion and recommendation:**

The willingness to utilize cervical cancer screening services is low in the Girar Jarso district. To improve community awareness and attitude, continued and sustainable advocacy on the value of cervical cancer screening should be offered through mass media and health extension workers.

## Introduction

Approximately 570,000 cases and 311,000 deaths from cervical cancer are reported each year worldwide, making it the leading cause of death and illness among women (1). Global trends show that, in developing countries going through rapid societal and economic changes, the shift toward lifestyles like that of industrialized countries leads to a rising burden of any type of cancer (2). There is a large difference between developing and developed countries, where cervical cancer cases have been significantly reduced since the implementation of effective screening programs (3). China's cervical cancer screening rate and willingness were found to be 35.1 and 82.22%, respectively (4).

In Africa, cervical cancer is the second highest type of cancer occurring among women with an estimated 119,284 new cases and 81,687 fatalities each year, showing an increasing trend (5). In developed countries, programs exist that allow girls to be vaccinated against HPV and women to get screened regularly and treated appropriately on a regular basis, whereas in low- and middle-income countries (LMICs), access to these preventative measures is limited, and the disease is often unappreciated until it has progressed to its final stage (6). Although there was a high level of readiness to have a cervical cancer screening, the rate of screening is still low. Sub-Saharan Africa, for instance, has a strong propensity to use screening (7, 8).

In Ethiopia, over 6,294 new cases and 4,884 deaths occur each year, making it the second greatest cause of mortality following breast cancer, and the lion's share was among women aged 30–65 years, accounting for 79% of new cases and 65.8% of deaths per year (5). The prevalence and fatality rate in Ethiopia could probably be significantly higher than what was reported in a meta-analysis study given the sparse amount of data currently available (9). HPV vaccination and cytological screening programs can prevent cervical cancer, but these services are insufficient in LMICs, particularly in sub-Saharan Africa, including Ethiopia, where the issues are still devastating due to a lack of resources and a weak healthcare system, making cervical cancer screening opportunistic rather than planned. The chance of developing cancer can be decreased by 25–36% in women between the ages of 30 and 40 years who undergo a cervical cancer evaluation one time in their life (2–4). According to a study conducted in several regions of Ethiopia, women in the Ethiopian army (43.4%), Debre Berhan Town (45.3%) (6), and Addis Ababa (62.7%) (7) expressed a willingness to get a cervical cancer screening.

According to research, Ethiopia's prevalence of cervical cancer screening for disease prevention is low, at around 2% nationwide (4). The Federal Ministry of Health launched visual inspection with acetic acid (VIA) screening in conjunction with access to cryotherapy (7) at 800 healthcare facilities in order to increase the screening coverage by more than 80%. Furthermore, not only was a cancer registry established in several areas of the country, including the study area, to lessen the effects of cervical cancer, to promote cancer surveillance, and to register and research cervical cancer but also media campaign was made many times and is still in progress. As a result, a total of 22,818 women between the ages of 30–49 years underwent cervical cancer screening in 2015 (4, 8).

Despite the efforts made to increase cervical cancer screening, the rate of screening is still low. As a result, the purpose of this research is to learn about the community's willingness in the study area. To the best of our knowledge, no research has been done on the willingness to utilize cervical cancer screening among women aged 30–65 years, where the disease is most prevalent. This study is the first in the Girar Jarso district to assess the prevalence of willingness to utilize cervical cancer screening among women aged 30–65 years and to identify factors associated with the discovered prevalence.

## Methods and materials

### Study design and setting

A community-based cross-sectional study was conducted in the Girar Jarso district of North Shoa zone, Oromia region, Ethiopia, from 1 June 2021 to 1 September 2021. All women between the ages of 30 and 65 years who lived in specified kebeles for at least 6 months were included in this study. Individuals who were seriously ill (unable to provide correct and required information) were not allowed to participate.

### Sample size and sampling technique

The sample size was calculated using the proportion of women willing to be screen for cervical cancer (62.7%) (7), a standard score of 95% CI and a 5% margin of error, and a 10% nonresponse rate. After adding the design effect, a total sample size of 790 was determined. A stratified cluster sampling technique was employed. The Girar Jarso district is divided into two sections, namely, urban and rural. Five rural clusters and one urban cluster were chosen at random from 21 kebeles, and all households within those clusters were included in the study. Finally, each home in each of the selected kebeles was interviewed consequently. When there was more than one eligible woman in a home, one was chosen at random using the lottery technique. Women between the ages of 30 and 65 years were interviewed in the selected kebeles. When an eligible participant was not at home, data collectors returned to the household at different time intervals or used a different method (i.e., phone calls after his/her first journey if available), and interviewers were given the option of collecting data after working hours and on weekends if they were unable to locate the eligible participant. If interviewers were unable to locate the eligible participant, the household was marked as a nonresponse.

### Data collection tools and procedures

Data were collected using an interviewer-administered questionnaire. A questionnaire was adopted from related literature (7, 8, 10), and it was translated into Afaan Oromo and then back to English to ensure consistency. Before real data collection, the translated questionnaires were pretested, and a few questions were modified after discussion with the study teams. Age, residence, marital status, religion, education status, occupation, income, and health insurance were among the sociodemographic data. Contraceptive use, history of STIs, sero-HIV status, age of sexual intercourse, parity, duration of the marriage, and history of gynecologic examination are among the reproductive-related factors. Cervical cancer screening attitudes, knowledge, and barriers to utilize were also included.

Willingness to be screened for cervical cancer is defined as a woman who is willing to be tested and has decided to be screened in the coming year but has never been screened previously (10). Those who had never screened for cervical cancer over their lives were regarded as never screened, while those who had screened at least one time were considered as screened. Eight attitude questions about cervical cancer and screening were presented and measured on a Likert scale (1 = “Strongly disagree”; 2 = “Disagree”; 3 = “Neutral”; 4 = “Agree”; and 5 = “Strongly agree”). Then, those who scored mean and above were categorized as having a “positive attitude” and those who scored below the mean were categorized as having a “negative attitude.” Eighteen knowledge questions about cervical cancer and screening were presented, and the correct answer scored 1 and the incorrect answer scored 0. The total points to be scored were 18, and the minimum was 0. Then, those who scored mean and above were categorized as having “good knowledge” and those who scored below the mean were categorized as having “poor knowledge.” Nineteen perceived barriers questions about cervical cancer and screening were presented and measured on a Likert scale (1 = “Strongly disagree”; 2 = “Disagree”; 3 = “Neutral”; 4 = “Agree”; and 5 = “Strongly agree”). Then, those who scored mean and above were categorized as having a “high perceived barrier” and those who scored below the mean were categorized as having a “low perceived barrier” (9). To examine the internal consistency of distinct items, Cronbach's alpha coefficients were calculated. Nineteen items with a Cronbach's alpha of 0.95 were used to assess perceived barriers. Attitudes were also measured by eight items with Cronbach's alpha of 0.800 and 0.76 for knowledge of cervical cancer and screening.

### Data processing and analysis

Data were double-checked, coded, and entered into EpiData version 4.6 before being exported to SPSS (Statistical Package for Social science) version 23 for analysis. Tables, graphs, and frequency were utilized to portray the data in the descriptive statistics tables. Binary logistic regression was fitted to identify factors associated with the willingness to utilize cervical cancer screening. Variables with a *p*-value of <0.25 were identified and fitted to multivariable logistic regression. Variables scoring a *p*-value of <0.05 at 95% CI were considered significant. Variance inflation factors (VIF) were utilized to screen for multicollinearity, and a VIF of <10 was employed as a cutoff point to diagnose multicollinearity. A *p*-value of >0.05 was used to indicate the model fit using the Hosmer and Lemeshow model's goodness-of-fit test.

## Results

### Background characteristics

In this study, 855 participants were involved; among them, 597 (60.9%) participants were from rural areas, and 383 (48.8%) participants were between the ages of 30 and 39 years. Approximately 643 (75%) respondents practiced the orthodox religion. Of the participants in the interview, seven hundred forty-seven (87.4%) were married, and among them, 443 (51.8%) were housewives. In total, four hundred ninety-six (58%) participants had health insurance. Of the female participants, two hundred eighty-six (33.5%), or roughly one-third of them, attended primary school. Among all respondents, 200 (23.4%) households were in the middle quintile in terms of wealth (Table 1).

**Table 1:** Sociodemographic characteristics of women aged 30–65 years in the Girar Jarso district, Ethiopia (*N* = 855) during January 2022.

**Variable**	**Frequency**	**Percentage (%)**
**Age group**
30–39	383	44.8
40–49	276	32.3
50–60	135	15.8
≥60	61	7.1
**Residence**
Urban	258	30.2
Rural	597	69.8
**Religion**
Orthodox	643	75.2
Protestant	151	17.7
Muslim	58	6.8
Catholic	2	0.2
Other^*^	1	0.1
**Marital status**
Single	12	1.4
Married	747	87.4
Widowed	45	5.2
Divorced	39	4.6
Separated	12	1.4
**Women educational status**		
Didn't read and write	274	32.0
Primary	286	33.5
Secondary	174	20.3
College and above	121	14.2
**Current occupation**
Housewife	443	51.8
Small business	248	29.0
Daily laborer	22	2.6
Government employee	110	12.9
Private employee	32	3.7
**Health insurance**
Yes	496	58.0
No	359	42.0
**Husband educational status**		
Didn't read and write	236	28
Primary	283	33.6
Secondary	141	16.7
College and above	183	21.7
**Wealth status**
Lowest	174	20.4
Second	167	19.5
Middle	200	23.4
Fourth	149	17.4
Highest	165	19.31

Other

* implies Waaqeffana.

### Reproductive characteristics

Of the 855 respondents, 485 (56.7%) got married before the age of 20 years, and 531 (63.5%) had at least one child, with 457 (55.6%) respondents using a modern form of contraception. An HIV test was administered to more than 800 people (87.5%), and 684 of them (81.5%) received a negative result; 591 (69%) participants had their first sexual experience before turning 20 years of age (Table 2).

**Table 2:** Reproductive characteristics among women aged 30–65 years in the Girar Jarso district, Ethiopia (*N* = 855) during January 2022.

**Variable**	**Frequency (*n*)**	**(%)**
**Age of first marriage**		
<20 years	485	56.7
≥20 years	370	43.3
**Birth delivery**
No	17	0.2
Yes	838	98.0
**Parity**
<3	532	63.5
≥3	306	36.5
**Mode of delivery**
Vaginal	751	89.6
Cesarean	48	5.7
Both	39	4.7
**Ever used modern contraceptive**		
Yes	475	55.6
No	380	44.4
**History of STI**
Yes	73	8.5
No	782	91.5
**HIV test**
Yes	800	87.5
No	55	12.5
**Self-reported HIV result**		
Positive	116	14.5
Negative	684	85.5
**Age of first sexual intercourse**		
<20 years	591	69.1
≥20 years	264	30.9

### Awareness of cervical cancer screening

**Figure 1:**
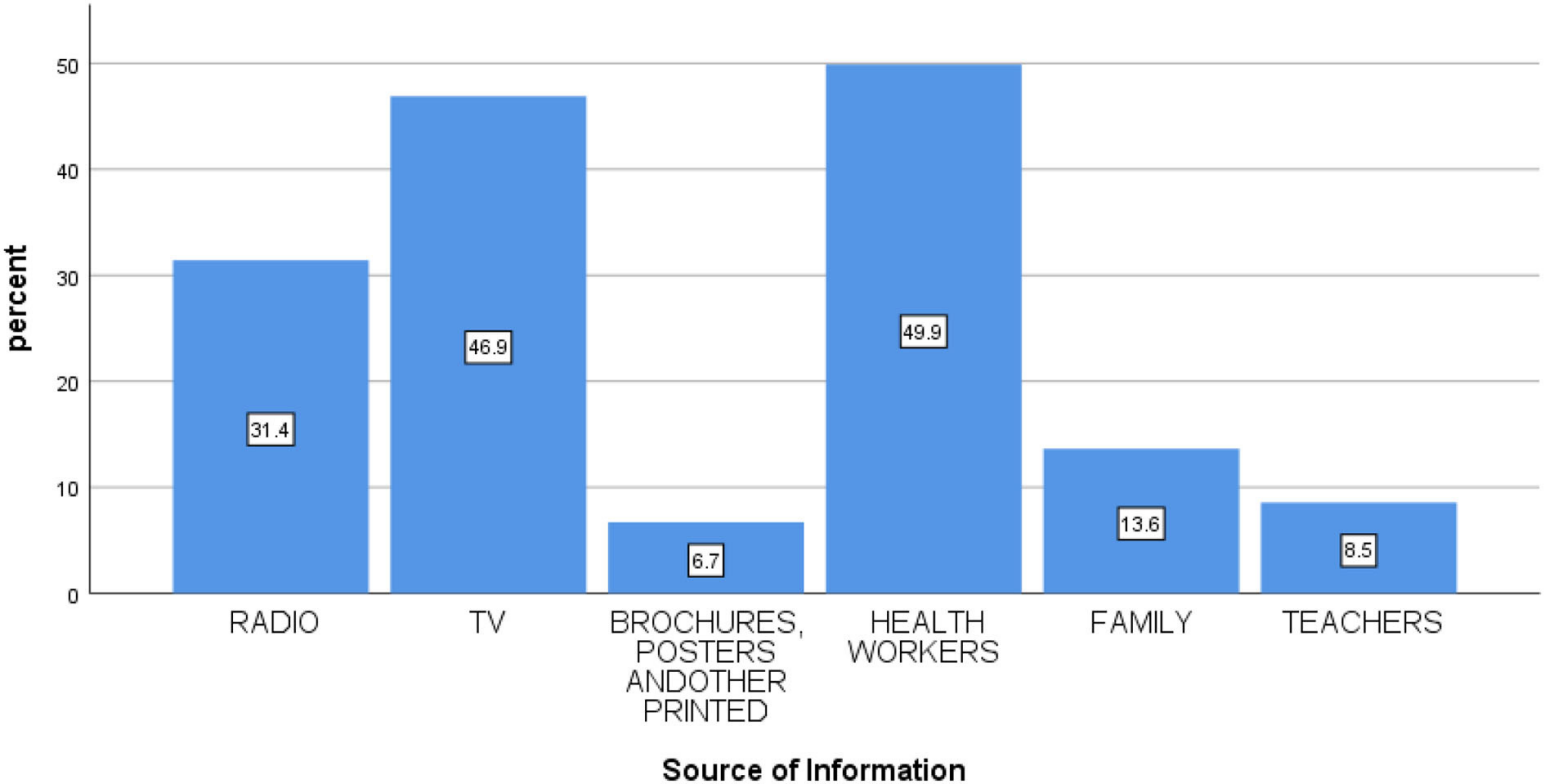
Source of information for cervical cancer screening.

Of the respondents, four hundred fifty-nine (53.7%) had heard about cervical cancer. Among those participants who heard about cervical cancer, half (459, 53.7%) of them responded that cervical cancer is preventable and one-fourth (247, 28.9%) of them reported that it is a communicable disease, whereas only 381 (44.6%) of the participants had heard about cervical cancer screening and only 289 (33.8%) of the participants knew where the screening center is located (Table 3). The primary source of information for the respondents was health workers (216, 48.9%), followed by television (203, 46.9%) (Figure 1).

**Table 3:** Awareness of cervical cancer screening among women aged 30–65 years in the Girar Jarso district, Ethiopia (*N* = 855) during January 2022.

**Awareness of cervical cancer screening**	**Frequency**	**Percentage (%)**
**Heard of cervical cancer**		
No	396	46.3
Yes	459	53.7
**Cervical cancer is curable**		
No	447	52.3
Yes	408	47.7
**Cervical cancer is preventable**		
No	396	46.7
Yes	459	53.3
**Heard of cervical cancer screening**		
No	474	55.4
Yes	381	44.6
**Awareness of where to screen**		
No	566	66.2
Yes	289	33.8
**Know someone screened for cervical cancer**		
No	737	86.2
Yes	118	13.8
**Know someone with cervical cancer**		
No	793	92.7
Yes	62	7.3

### Knowledge of cervical cancer and screening

**Figure 2:**
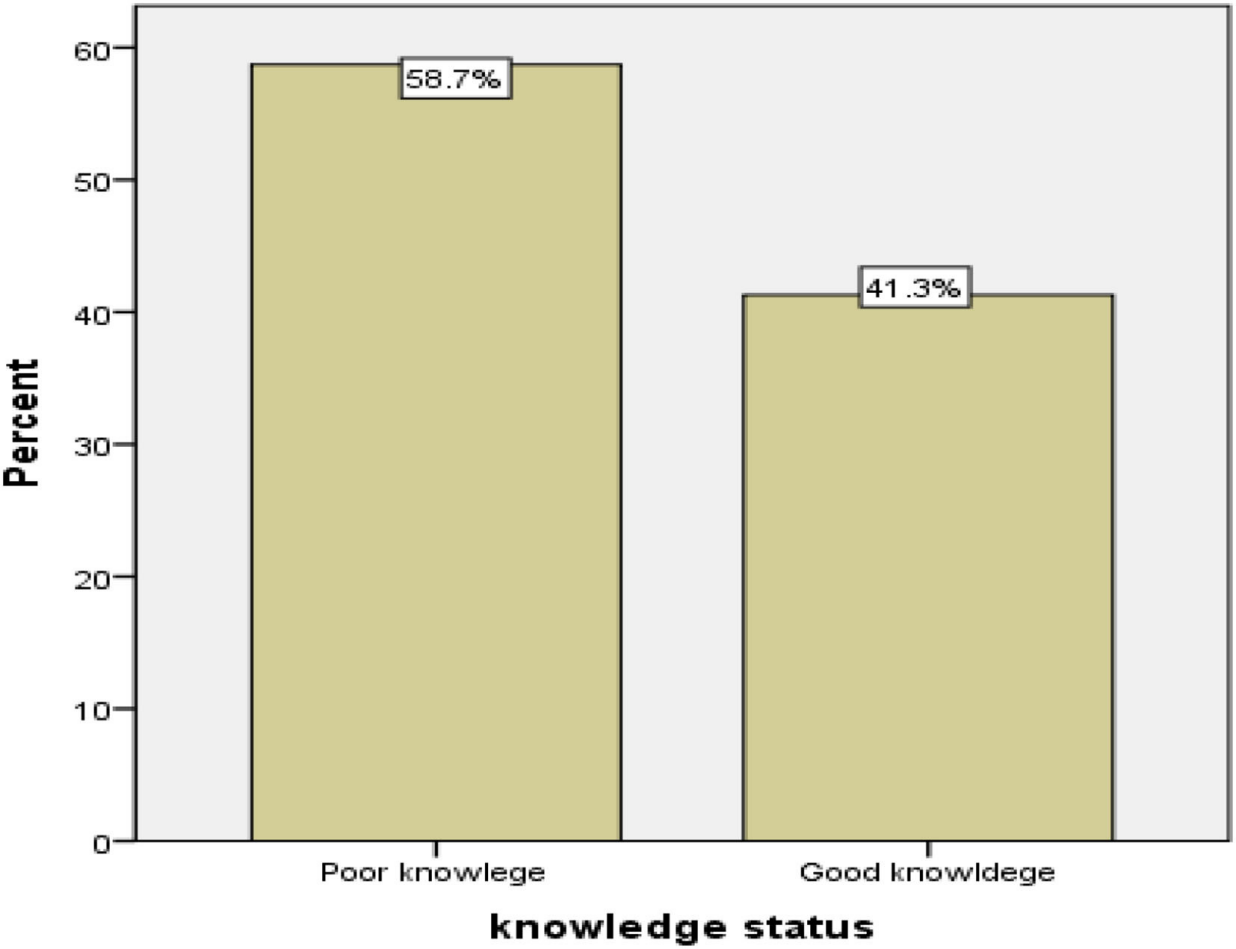
Knowledge of cervical cancer and screening service utilization among women aged 30–36 years in the Girar Jarso district, Ehiopia during January 2022.

Among those who ever heard about cervical cancer, only 353 (41.3%) participants had good knowledge. Only 125 (33%) and 123 (24.5%) of those with good knowledge were willing to use and had utilized cervical cancer screening at least one time in their lives, respectively (Figure 2).

### Attitude and barriers toward cervical cancer screening

Of the current respondents, four hundred seventy-four (55.4%) had a positive attitude, and two-thirds (65.4%) of those who had a good attitude were willing to be examined for cervical cancer. Of all the study participants, 455 (53.2%) had low perceived barriers, and nearly two-thirds (65.1%) of those respondents were willing to uptake cervical cancer screening.

### The prevalence of willingness to utilize cervical cancer screening

Only 315 (46.7%) respondents who had never screened for cervical cancer were willing to do so, whereas only 181 (21.2%) respondents had been screened for cervical cancer at least one time in their lives. Women were unwilling to be examined for cervical cancer because they felt healthy (73.7%), were shy, were afraid of a positive result, did not know about the service, believed that screening was expensive, and did not have permission from their husbands.

### Factors associated with the willingness to utilize cervical cancer screening

The results of the bivariable and multivariable logistic regressions are documented in Table 4. In a b-ivariable analysis, place of residence; age; current occupation of women; husband's educational status; having health insurance; the age of first marriage; parity; modern contraceptive use; duration of contraceptive use; self-reported HIV test; the age of first sexual intercourse; aware that cervical cancer is preventable, communicable, and killer; perceived barriers; wealth status of the respondent; attitude; knowledge about cervical cancer; and screening were found to be associated with the willingness to utilize cervical cancer screening (*p* < 0.25).

**Table 4:** Factors associated with the willingness to utilize cervical cancer screening service among women aged 30–65 years in the Girar Jarso district, Ethiopia during January 2022.

**Variable**	**Willingness to CCS**		**COR (95%)**	**AOR (95%)**
	**Yes**	**No**		
**Age of respondent**				
30–39	172	129	4.13 (1.95, 8.74)	2.80 (1.05, 7.48)^*^
40–49	92	132	2.16 (1.10, 4.62)	1.71 (0.66, 4.48)
50–59	41	67	1.89 (0.84, 4.27)	1.59 (0.58, 4.32)
≥60	10	31	1	
**Residence**				
Rural	143	40	1	
Urban	172	319	6.63 (4.46, 9.86)	2.12 (1.06, 4.48)^*^
**Husband's educational status**				
Unable to read and write	74	110	1	
Primary	91	154	0.88 (0.59, 1.30)	0.69 (0.42, 1.14)
Secondary	55	63	1.29 (0.81, 2.06)	0.56 (0.30, 1.03)
College and above	92	24	5.69 (3.33, 9.75)	0.57 (0.23, 1.41)
**Current occupation**				
Housewife	139	216	1	
Merchant	94	121	1.23 (0.86, 1.70)	0.65 (0.41, 1.03)
Daily laborer	15	6	3.8 (1.47, 10.25)	1.07 (0.31, 3.62)
Government employee	50	10	7.8 (3.81, 15.83)	1.49 (0.58, 3.81)
Private employee	17	6	4.4 (1.69, 11.43)	1.58 (0.39, 6.51)
**Health insurance**				
No	115	157	1	
Yes	200	202	0.74 (0.54, 1.01)	1.44 (0.96, 2.15)
**Age of first marriage**				
<20 years	155	260	1	
≥20 years	160	99	2.71 (1.96, 3.73)	1.61 (0.90, 2.89)
**Parity**				
<3 children	228	185	1	
≥3 children	84	167	0.41 (0.29, 0.56)	1.17 (0.71, 1.91)
**Modern contraceptive use**				
No	121	189	1	
Yes	194	170	1.78 (1.31, 2.42)	1.33 (0.59, 2.99)
**Duration of contraceptive use**				
<6 years	157	154	1	
≥6 yeeras	158	205	0.76 (0.56, 1.02)	1.14 (0.52, 2.49)
**History of HIV test**				
No	7	28	1	
Yes	308	331	3.7 (1.60, 8.64)	1.97 (0.71, 5.46)
**Age of first intercourse**				
<20 years	208	290	1	
≥20 years	107	69	2.16 (1.52, 3.07)	0.74 (0.38, 1.41)
**Cervical cancer is preventable**				
No	97	246	1	
Yes	218	113	4.89 (3.53, 6.78)	3.41 (1.41, 8.23)^*^
**Cervical cancer is killer**				
No	47	164	1	
Yes	268	195	4.79 (3.30, 6.96)	2.67 (1.65, 4.33)^*^
**Cervical cancer is communicable**				
No	223	294	1	
Yes	92	65	1.86 (1.29, 2.68)	1.85 (1.09, 3.13)^*^
**Knowledge of cervical cancer**				
Poor knowledge	190	105	1	
Good knowledge	125	254	0.27 (0.19, 0.37)	1.58 (0.68, 3.65)
**Attitude**				
Negative	109	207	1	
Positive	206	152	2.57 (1.88, 3.52)	1.68 **(**1.11, 2.53)^*^
**Perceived barrier**				
Low	205	117	3.85 (2.79, 5.32)	1.91 (1.25, 2.91)^*^
High	110	242	1	
**Wealth quintile**				
Lowest	33	123	1	
Second	44	95	1.72 (1.02, 2.92)	1.39 (0.77, 2.52)
Middle	75	90	3.11 (1.90, 5.08)	2.16 (1.23, 3.77)^*^
Fourth	76	22	12.87 (6.99, 23.71)	3.62 (1.49, 8.80)^*^
Highest	87	29	11.18 (6.32, 19.76)	3.19 (1.29, 7.91)^*^

CCS, cervical cancer screening, 1 = reference, and

* Statistically significant at *p* < 0.05 in bivariable and multivariable logistic regressions.

In a multivariable analysis, women's age, residence, attitude, awareness of cervical cancer, perceived barrier, and wealth status of the respondent were found to be independently significant. All women aged 30–49 years were 2.8 times more willing to be screened for cervical cancer as compared with those ≥60 years (AOR = 2.80; 95% CI: 1.05, 7.48). Urban residents were 2.12 times more willing for cervical screening compared with rural residents (AOR = 2.12; 95% CI: 1.06, 4.48). Women who were aware that cervical cancer is preventable had an AOR of 3.41; 95% CI: 1.41, 8.23, women who were aware that cervical cancer is a killer had an AOR of 2.67; 95% CI: 1.65, 4.33, and women who were aware that cervical cancer is communicable had an AOR of 1.85; 95% CI: 1.09, 3.13. Women who had a positive attitude toward the willingness to utilize cervical cancer screening were 1.68 times more likely compared with those who had a negative attitude (AOR = 1.68; 95% CI: 1.11, 2.53). Women who had low perceived barriers toward cervical cancer screening were two timesmore willing compared with those who had high perceived barriers (AOR = 1.91; 95% CI: 1.25, 2.91). Finally, factors such as women who were in the middle (AOR = 2.16; 95% CI: 1.23, 3.77), fourth (AOR = 3.62;95%CI:1.49,8.80), and high estquintile (wealthiest) [AOR = 3.19;95%CI:(1.29,7.91)] were found to increase willingness to utilize cervical cancer screening.

## Discussion

This study aimed to assess the willingness to utilize cervical cancer screening among women aged 30–65 years in the Girar Jarso district, Ethiopia. Accordingly, the prevalence of willingness to utilize cervical cancer screening was found to be 46.7% (95% CI: 43, 50.3).

Our results were consistent with the research done among Ethiopian army women (43.4%) (11) and among women in Debre Berhan town (45.3%) (6), where screening for cervical cancer was offered to participants. However, this result was lower than that of studies conducted in Addis Ababa, Ethiopia (62.7%) (7); Nigeria [three studies−73.6% (10), 86.7% (12), and 88.9% (13), respectively]; Mozambique (86%) (14); and South America (88.9%) (15) among Latino immigrants. This discrepancy may be explained by a number of sociodemographic factors, including a person's perception of their own health or their ignorance of the services offered by a health facility. In fact, nearly a third of our respondents (73.7%) expressed feeling healthy and their reluctance to get an examination. Additionally, 58.7% of participants had inadequate knowledge, and 18.9% of women were unaware of the service. The outcome of this study, however, was higher than those of two studies done in India [32% (16) and 20% (17), respectively]. This discrepancy may be attributed to varying study populations, study periods, and cultural variables that make it challenging for the community to accept cervical cancer screening.

Women aged 30–39 years were found to be more willing to be screened for cervical cancer than women aged ≥60 years. This conclusion was consistent with a Tanzanian study that found younger age (30–39 years) was substantially associated with the willingness to screen (11) when compared with other age groups. This could be related to the high-level of education attainment. In fact, those aged 30–39 years had a higher educational attainment level (21.1%) than those aged 60 and above years (7.1%). As the 30–39 age group represents the reproductive age for most women in a resource-constrained environment, the high readiness to screen for cervical cancer may be attributable to more frequent exposure to reproductive services and care than women in other age groups. Furthermore, the cervical cancer screening guideline in Ethiopia encourages women aged 30–49 years to be screened for cervical cancer, and women in this age group may have better knowledge and intention to be screened than women in other age groups (12). Moreover, this age group is a productive age with the possibility of receiving more gynecological examinations, giving birth, and receiving more health information about reproductive health, including cervical cancer, which promotes screening services and may give more readiness to receive screening.

To rule out the contribution of the current address on the willingness to screen for cervical cancer, we included sociodemographic characteristics in our model. According to our findings, participants in the urban area were 2.12 times more willing to be examined than in the rural area. Our findings were compared with those reported in a Tanzanian study (11). One possible explanation is that women who live in cities have easy access to information from various sources such as the media and health centers. This argument was reinforced by a Ugandan study that found that women who lived in urban areas had a higher knowledge of cervical cancer (13). Women who live in cities have easier access to healthcare than women who live in rural areas. Residing in a rural area is a risk factor because cervical cancer screening in Ethiopia is done by a few public hospitals that are mainly located in urban areas.

The wealth status of the study participants was also another strong predictor of the willingness to utilize cervical cancer screening services. In comparison with those in the lowest quintile, women in the middle, fourth, and highest quintiles were substantially related to their willingness to get screened for cervical cancer. This report was consistent with the study report from Ethiopia (14), Tanzania (15), and two studies from China (4, 16). These findings might be unsurprising because people of high socioeconomic class are likely to be concerned about their health even before they show signs and symptoms and they are unconcerned about the cost of screening. They can also travel long distances for screening by transportation. This may be because those having more income have additional money to allocate for the promotion of their health in addition to meeting their basic needs. Studies conducted in Addis Ababa (17), Malaysia (18), Iran (19), and Korea (20) for different healthcare services support this conclusion. There was a result that people with a higher income were more aware of cervical cancer, implying that there was a link (21).

According to a study from Addis Ababa, Ethiopia (7), and Nigeria (10), there was a substantial link between cervical cancer awareness and the willingness to utilize cervical cancer screening. Similarly, in this study, participants who were aware that cervical cancer is communicable, killer, and preventable were found to be substantial predictors of the willingness to utilize cervical cancer screening compared with those who were unaware. This could be explained by participants who were aware of cervical cancer in general, meaning that those who reported cervical cancer as a killer, communicable disease, and preventable disease were concerned, fearful, and wanted to be screened. Another possible explanation is that a respondent who is aware is more likely to be aware of the benefits and choose it. It is possible that public awareness is being raised as a result of a mass media campaign focusing on a certain component of the problem.

When compared with women who had a negative attitude toward cervical cancer screening, those who had a positive attitude were found to be substantially related. Similarly, we observed that attitudes regarding cervical cancer screening were important determinants affecting willingness in reports from the United States (22), Canada (23), and Ethiopia (6). The likely rationale is that participants with a favorable attitude regarding cervical cancer screening may be able to change thoughts and minimize or discard reluctance of their own beliefs, increasing the likelihood of being willing to utilize cervical cancer screening.

Another variable that predicted the willingness to utilize cervical cancer screening was the perceived barrier. Women who thought there were low barriers to cervical cancer screening were more likely to use it than those who thought there were many. This finding is compared with a recent study in China (24) and Uganda (25), which found that a personal barrier, such as feeling apprehensive if the screening revealed discomfort, could be a negative inducement for screening attendance. Other barriers include social barriers such as fear of being screened because it is considered immoral, not being recommended by family to undergo screening, of fear of others learning their results, and husband's refusal to allow, as well as facility barriers such as preferring female doctors, not having a health facility close to home, and having to wait a long time to have screening. Low barrier denotes that there is a low facility barrier or a high density of health institutions, and satisfactory outreach activities are held in the district. This could be explained by women's view of their own vulnerability to illness, and if they perceived that they are prone to cancer of the cervix, they seek screening and medical care to protect themselves.

## Strengths and limitations of the study

We identified different factors associated with the willingness to utilize cervical cancer screening, including the rural community, which makes it the first study of its kind because most previous studies focused on urban institutions and making population inferences. On the contrary, some factors may be subject to social desirability bias; however, we trained female data collectors to eliminate this as much as possible. Due to its cross-sectional character, it does not show a causal relationship.

## Conclusion

The willingness of women aged 30–65 years to utilize cervical cancer screening was found to be low in this study. Age; residing in a city; knowing that cervical cancer is preventable, killer, and communicable; having a positive attitude; having a low perceived barrier; and being in the middle, fourth, or highest quintile were all predictors of the willingness to utilize cervical cancer screening programs. As a result, through various media and health extension workers, efforts should be made to raise community awareness and attitude. Furthermore, to influence community beliefs about cervical cancer screening, a community-based screening program should be established and implemented with the help of key national partners and organizations.

## Data availability statement

The original contributions presented in the study are included in the article/supplementary material, further inquiries can be directed to the corresponding author/s.

## Ethics statement

Letter of ethical clearance and approval was obtained from Salale University Ethical Review Committee. The patients/participants provided their written informed consent to participate in this study.

## Author contributions

All authors made a significant contribution to the conception, study design, execution, acquisition of data, analysis, and interpretation. All authors were involved in drafting, revising, or critically reviewing the article, gave final approval of the version to be published, have agreed on the journal to which the article has been submitted, and agreed to be accountable for all aspects of the work.

## Conflict of interest

The authors declare that the research was conducted in the absence of any commercial or financial relationships that could be construed as a potential conflict of interest.

## Publisher's note

All claims expressed in this article are solely those of the authors and do not necessarily represent those of their affiliated organizations, or those of the publisher, the editors and the reviewers. Any product that may be evaluated in this article, or claim that may be made by its manufacturer, is not guaranteed or endorsed by the publisher.

## Abbreviations

AOR: adjusted odds ratio
CCS: cervical cancer screening
COR: crude odds ratio
HIV: human immunodeficiency virus
HPV: human papillomavirus
LMICs: low and middle-income countries
STI: sexually transmitted infection
VIA: visual inspection with acetic acid
VIF: variance of inflation factor.
